# Parametrical modelling for texture characterization—A novel approach applied to ultrasound thyroid segmentation

**DOI:** 10.1371/journal.pone.0211215

**Published:** 2019-01-29

**Authors:** Alfredo Illanes, Nazila Esmaeili, Prabal Poudel, Sathish Balakrishnan, Michael Friebe

**Affiliations:** INKA, Institute of Medical Technology, Otto-von-Guericke-Universität Magdeburg, Magdeburg, Germany; University of California Los Angeles, UNITED STATES

## Abstract

Texture analysis is an important topic in Ultrasound (US) image analysis for structure segmentation and tissue classification. In this work a novel approach for US image texture feature extraction is presented. It is mainly based on parametrical modelling of a signal version of the US image in order to process it as data resulting from a dynamical process. Because of the predictive characteristics of such a model representation, good estimations of texture features can be obtained with less data than generally used methods require, allowing higher robustness to low Signal-to-Noise ratio and a more localized US image analysis. The usability of the proposed approach was demonstrated by extracting texture features for segmenting the thyroid in US images. The obtained results showed that features corresponding to energy ratios between different modelled texture frequency bands allowed to clearly distinguish between thyroid and non-thyroid texture. A simple k-means clustering algorithm has been used for separating US image patches as belonging to thyroid or not. Segmentation of thyroid was performed in two different datasets obtaining Dice coefficients over 85%.

## Introduction

Texture analysis is the term used for methods developed to quantify image texture through description of image properties by textural features. In general, these features aim to measure smoothness, coarseness, and regularity of pixels, which form an image [1, 2]. Feature extraction methods are usually followed by classification or clustering and can be applied for image segmentation, image characterization, and for estimation of image similarity metrics [3].

Generally used approaches for computing texture features are based on statistical and frequency domain techniques. Statistical approaches compute histograms, entropy, homogeneity, mean and variance values for estimating features from the texture. Frequency domain techniques or spectral techniques collect a distribution of filter responses to extract different aspects from the texture [1]. Gabor filters and Wavelet decomposition are examples of this type of approach.

The main drawback of these approaches is that they are mainly data-driven, meaning that the computation of texture characteristics is performed directly from the pixel values. With that the estimation of texture characteristics is limited by the amount of data and the Signal to Noise Ratio (SNR) of the image.

In medical imaging, texture describes internal structures of human tissues or organs or pathological changes. Different modalities such as Magnetic Resonance, Computer Tomography and Ultrasound (US) require texture analysis and characterization for applications such as segmentation, registration ans lesion classification [3]. From all these modalities, US is known to be the most challenging because of the presence of characteristic artifacts such as speckles and shadows as well as due to the low SNR and resolution.

In this work, a novel approach for image texture feature extraction in US images is presented mainly based on parametrical modelling. The main idea behind this approach is to analyze the texture as data resulting from a dynamical process and to estimate the different dynamics involved in the texture in order to use mathematical operations between these dynamics as possible texture features. For that a signal version of the image is first computed, where the independent variable is the space, then the signal is decomposed in different frequency bands using Wavelet Transformation and finally an Autoregressive (AR) parametrical model of the decomposed signals provides spectral characteristics used for features computation.

The main advantage of this approach is that good estimations of texture characteristics can be obtained with less data due to the predictive characteristics of the model representation. Moreover, using an appropriate model order noise can be optimally handled and a better estimation of the dynamical properties of the texture can be obtained, even under really complex SNR characteristics as usually seen in US data.

The usability of the proposed approach was demonstrated with US data for segmenting thyroid texture. The obtained results showed that features corresponding to energy ratios between different modelled texture frequency bands allow to clearly distinguish between thyroid and non-thyroid texture regions.

The thyroid is one of the largest endocrine glands in the human body and it is involved in several significant body mechanisms. Diseases of the thyroid gland are among the most frequent endocrine disorders and changes of the thyroid volume are often the symptom of these common pathological conditions. For this reason, it is essential to track and monitor changes on thyroid volume over time and segmentation of the thyroid is one of the main steps for this purpose.

Many approaches have been presented in the literature for extracting features in US thyroid image analysis, mainly for thyroid segmentation and nodule characterization and classification. Recent surveys demonstrate that these two topics for thyroid analysis are highly active research fields [4–7]. Following this trend, many new methods have been proposed in the last years. Concerning thyroid segmentation in [8] three semi-automatic algorithms based on general segmentation approaches such as active contours, graph cut and pixel based classifier were evaluated and compared with two machine learning approaches based on Convolutional Neural Networks and Random Forest (RF). In [9] the segmentation of the thyroid is made by taking into account apriori information based on the physics of the US imaging process and by applying Iterative Random Walks and RF based techniques. Furthermore, several type of features have been proposed for tissue characterization in order to classify nodules or lesions in thyroid US images. Among the most used features are statistical features [10–12], Spectral-based features [13, 14], higher order statistics based features [15, 16], Wavelet-based features [2, 17, 18] and Fractal-based features [13, 19]. Additionally other works have proposed machine learning algorithms [20, 21] and neutrosophic clustering for thyroid tissue characterization.

As in the general literature for US feature extraction, the main drawback in thyroid US feature computation is that most of the proposed approaches are data driven operating directly from the pixel values of the image. We propose a completely different approach where the preprocessing or image aspects decomposition is made over a signal and not an image and where the features are computed not from the pixels values but from a parametrical model of each estimated image aspect. We believe that the predictive characteristics of such parametrical approach will be able to better deal with the low SNR of thyroid US images and will also allow to obtain better estimation of features with lower quantity of data than direct pixel feature computation.

The main purpose of this paper is not to propose a new thyroid segmentation algorithm but to show how features computed with a completely novel approach can be valuable for US texture characterization. However, to assess the performance of the proposed approach the algorithm was evaluated using two thyroid datasets obtaining Dice coefficients higher than 85% in both databases. Additionally, the results were compared with the ones obtained by other approaches proposed in the literature.

## Methods

As mentioned above, one of the major issues with US imaging is the quality of the data, which particularly affects segmentation applications or texture characterization that are strongly influenced by the relatively low quality of clinical US images, causing that tissue echogenic characteristics and boundaries are often drowned in noise. Decomposition and parametrization of each characteristic *aspect* of the data could reduce the noise and enhance the valuable information. These *aspects* could correspond to different type of noises or artifacts or to different levels of irregularity or granularity in the US image.

The core idea presented in this paper is to treat an US image as texture that can be represented as data resulting from a dynamical process, which depends on space as an independent variable and whose dynamical patterns can then characterize such a texture. These dynamics can be modelled using a parametrical approach and the estimated parameters can be taken as a mathematical representation of the texture that are used to compute valuable features that characterize the US texture at a given location.

**Fig 1:**
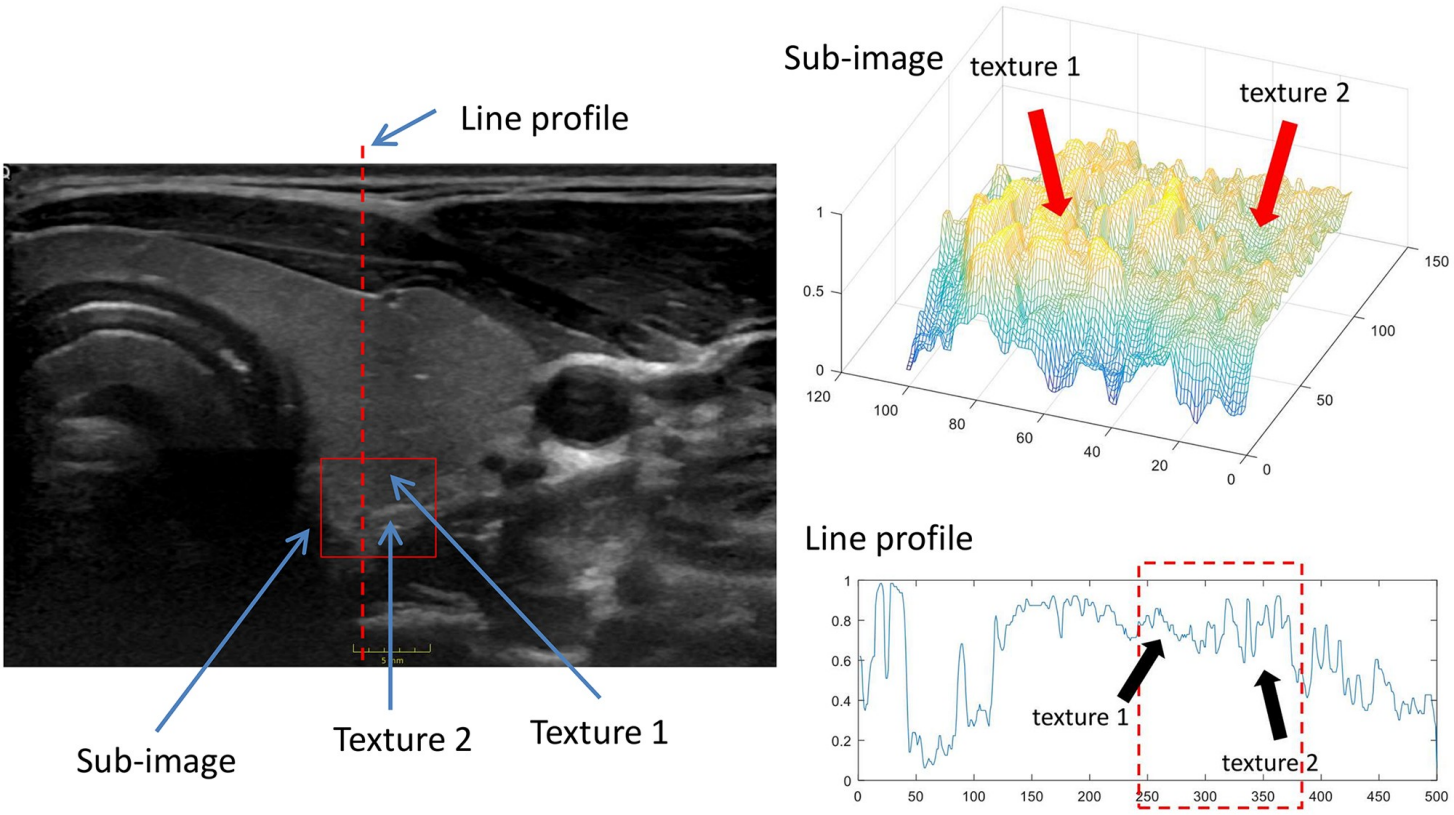
Illustration of the main principle behind the proposed US texture characterization approach.

Fig 1 illustrates the basic idea. A thyroid ultrasound image is shown and a sub-image or patch (red box) is selected in such a way that it contains thyroid (Texture 1 in the figure) and non-thyroid textures (Texture 2 in the figure). The boundary between the two textures (thyroid and non-thyroid) is not evident but in the mesh representation of the sub-image on the top right of Fig 1 it is possible to visualize the different texture characteristics of the two tissue types. If we extract a line profile passing through both textures (red dashed line in the US image) then it is possible to verify that the texture signals (inside the dashed rectangle over the line profile plot) involve different frequency components or more general, different signal dynamics that are characteristics of each texture.

The idea is now to model these texture dynamics using a parametrical approach to perform features computation not by operating the matrix data itself, but by operating the parameters of the modelled texture that represent the information that the image contains in terms of dynamical distribution. By using an optimal model order such an approach can be highly robust to the typical speckle noise of US images as well as to low trend intensity inhomogeneity. Additionally, because of the predictive characteristics of such a model representation, good estimations of characteristics of a texture can be obtained with less data than with standard methods.

**Fig 2:**
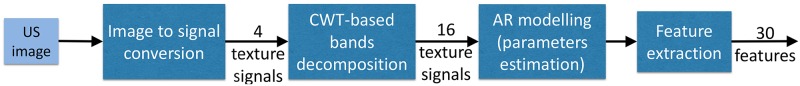
Main steps of the general concept of the signal processing algorithm for texture modelling and feature extraction in US images.

The block diagram of Fig 2 displays the main steps for feature computation of a US image patch in order to characterize its texture. First, the image patch gray-level matrix is converted into four texture signals using two different image to signal conversion procedures. Then, each of the texture signals is decomposed in four signal bands using Continuous Wavelet Transformation (CWT). The 16 resulting narrow-band texture signals are then modelled using an Autoregressive (AR) parametrical model to finally compute features from ratios between different energy bands of the decomposed signals. In the following, each step of the algorithm will be detailed.

### Image to signal conversion

**Fig 3:**
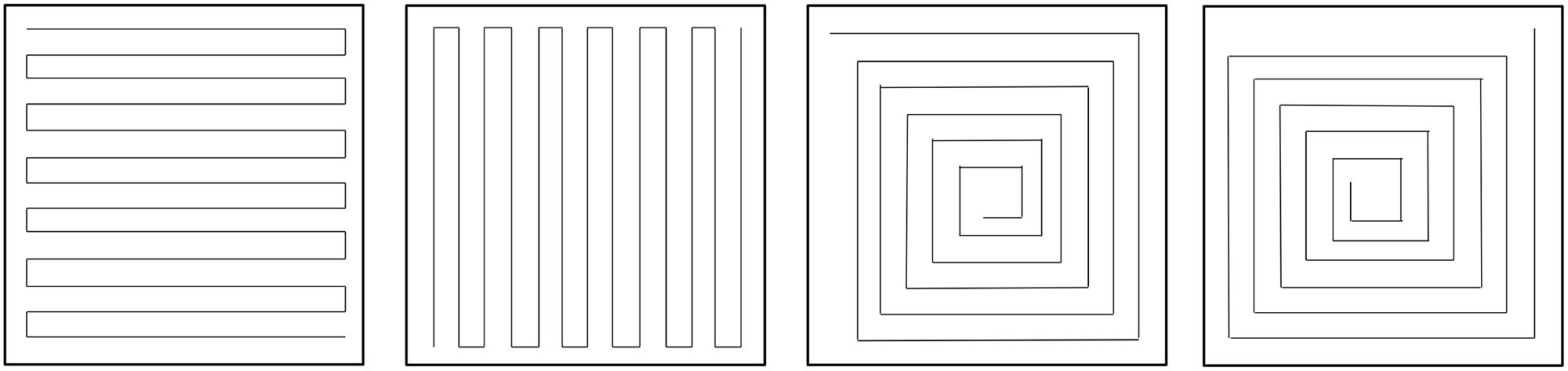
Conversion of a matrix by traversing the matrix and its transposed in ZigZag and in spiral.

In order to track dynamical texture characteristics of an US image resulting from a dynamical process, the matrix data is first converted into a signal. For that we use ZigZag (following the rows direction) and spiral conversion of the US matrix image and of their 90 degrees rotation matrix versions (see Fig 3. The output of this first step results in four texture signals, one per each conversion (ZigZag, spiral and their 90 degrees conversions).

### Continuous wavelet texture frequency band decomposition

The second step decomposes each one of the four texture signals in several frequency bands, each containing one different aspect of the texture. We assume that an image texture is composed of several dynamics representing irregularity characteristics of the texture such as smoothness or roughness. Therefore the signals can be decomposed into several dynamics that can represent levels of irregularities presented in the image/texture. Each signal is separated in different frequency components or scales and we then reconstruct several narrow band signals that should contain information of different levels of texture irregularity.

Since the four signals resulting from the image to signal conversion step can contain components that are not necessarily oscillatory, they are decomposed using scale decomposition instead of frequency Fourier- based decomposition. For that the CWT was applied to decompose the signal in different scales and then reconstructing new signals using scales equivalent to three frequency bands representing low, middle and high frequency components (LF, MF and HF) using a Daubechies mother wavelet. Additionally a fourth frequency band called Total Band (TB) was computed using the full frequency band of the signals but erasing the Very Low Frequency components Wavelet scales, which correspond to low trend image intensity inhomogeneity.

In summary this step results in 16 texture narrowband signals, four per texture signal. They are denoted in the sequel as $y_{i}^{B}\left[n\right]$, where *n* represent the discrete index (independent variable), *i* = 1, 2, 3, 4 denotes the signal conversion type (1, 2 for ZigZag and its rotated version, 3, 4 for spiral and its rotated version) and *B* denotes the type narrowband signal LF, MF, HF or TB.

**Fig 4:**
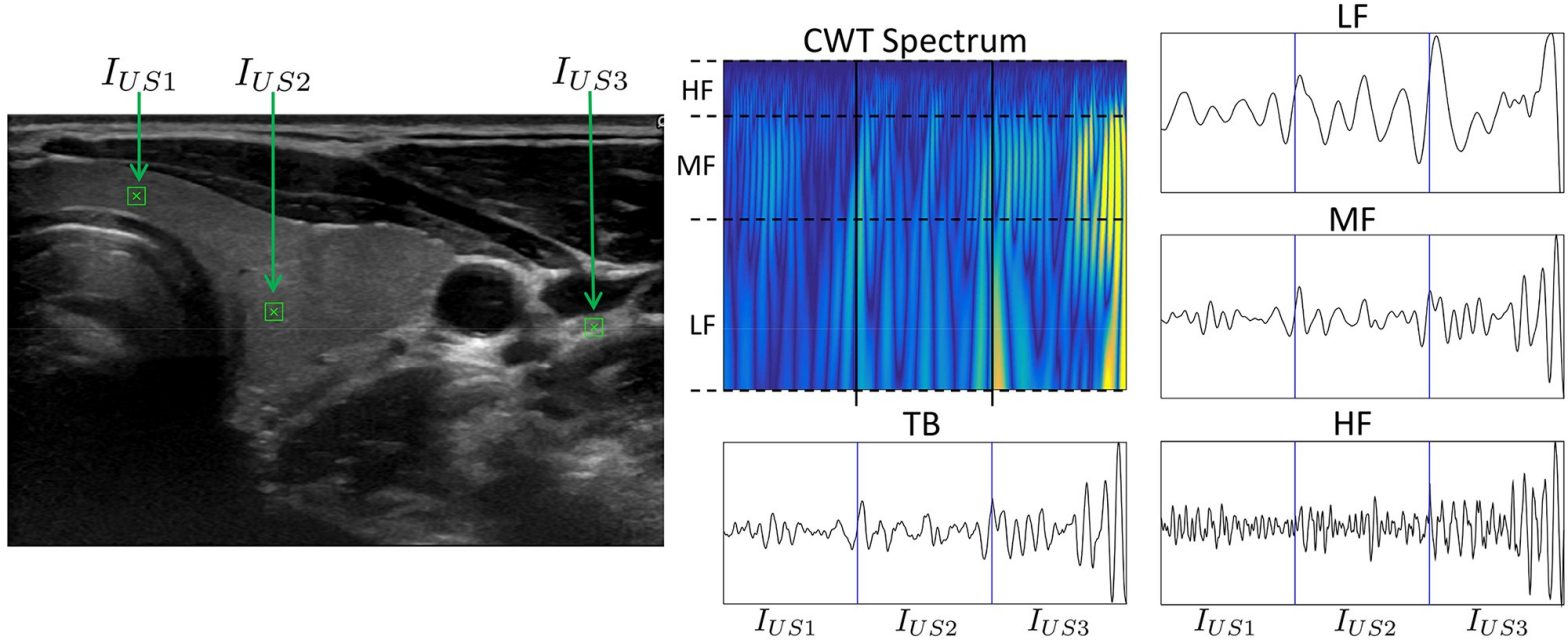
Example of a CWT decomposition of a thyroid US image when three image patches are taken from different locations of the US image.

Fig 4 shows an example of CWT decomposition of three texture signals (ZigZag non rotated conversion versions) at different positions in the US image denoted *I*_*US*1_, *I*_*US*2_ and *I*_*US*3_ in the figure. Two patches, *I*_*US*1_ and *I*_*US*2_, were taken from inside the thyroid and the patch *I*_*US*3_ was taken outside the thyroid, but in a region with similar texture characteristics as thyroid. At the right side of Fig 4 the CWT spectrum concatenated for the three patches is displayed. It is possible to visualize in the spectrum the scale frequency bands that were taken for constructing the narrow-band texture signals HF, MF and LF.

The TB, HF, MF and LF bands for the ZigZag signal version for each patch are shown also at the right of Fig 4 by concatenating the resulting signals in order to observe the dynamical difference between textures at different image locations. It is possible to observe that for *I*_*US*1_ and *I*_*US*2_, belonging both to patches located inside the thyroid, the dynamics of the decomposed signals are similar in MF, HF and TB, while the dynamics resulting from *I*_*US*3_ are clearly different. Particularly in HF it is evident that the frequency components and the amplitude are different inside compared to outside the thyroid. In LF even if the difference is low we can observe tiny changes in amplitude when we compare inside and outside thyroid patches. Given this analysis, what we want is to quantify these dynamical differences between image textures inside and outside the thyroid, and as explained in the next section, this will be done using a parametrical model of the different extracted texture signals.

### Ultrasound texture parametrical modelling

The size of a patch should be small enough in order to perform highly localized texture feature characterization. This requirement results in two characteristics of the data that a texture characterization method must deal with: the small quantity of dynamical changes (texture variability involving limited number of oscillations or damped oscillations) and the under-sampled characteristics of the data due not only to the size of the patch, but also to the resolution of the US image modality. Moreover, a texture characterization method should also be able to deal with the low SNR characteristics of US imaging. Under these conditions, classical methods for feature extraction, such as spectral or statistical based ones and in general data-driven approaches, no longer can obtain a good estimation of texture characteristics. This is why we propose in this work to use parametrical modelling of the resulting 16 texture signals from the CWT decomposition. These signals present narrow-band characteristics that are well suited to be modelled with an autoregressive (AR) approach. Our approach consist of a parametric representation of signal dynamics, which can deal with the drawbacks of the generally used current methods.

AR modelling is a well-known and well published technique for parametrical spectral estimation that has shown advantages over non-parametrical based methods (for detailed information about AR modelling we suggest [22]). The advantages of the AR representation is that it is possible to obtain good estimation of the spectrum and higher spectral resolution using less data than classical methods and that it provides a parametric way to analyze the data.

The AR model for each one of the sixteen $y_{i}^{B}\left[n\right]$ texture signals consists of a linear combination of past samples of the respective signal and a white zero mean noise *e*[*n*] of variance *σ*^2^:

$$\begin{array}{c} y_{i}^{B}\left[n\right]=-\sum_{k=1}^{p_{B}}a_{ik}^{B}y_{i}^{B}\left[n-k\right]+e\left[n\right] \end{array}$$ (1)

where $a_{ik}^{B}\left\{k=1,2,\ldots ,p_{B}\right\}$ are the estimated AR parameters for the narrowband signal $y_{i}^{B}\left[n\right]$. In this work the model order *p*_*B*_ is dependent on the band *B* and was set on 100, 50, 30 and 80 respectively for *B* = *LF*, *B* = *MF*, *B* = *HF* and *B* = *TB*. The AR parameters were estimated using the *Yule-Walker* method [22].

### Feature extraction and selection procedure

From Eq (1) power spectral densities can be computed for each one of the 16 narrowband signals. Since the textural dynamics should be different from one type of tissue to another, the main estimated components of the spectrum should vary between different tissues. Additionally, the different degrees of texture irregularities in a given tissue should be manifested by different contributions on energy of the frequency bands characterizing the texture signal. Therefore features related to spectral energy of the 16 texture signals (resulting after CWT decomposition) are used in this work.

To compute spectral energy features the Power Spectral Densities $S_{i}^{B}\left(f\right)$ for each one of the 16 narrowband signals have to be first computed from the AR parameters. For that the Z-transform can be applied to Eq (1) and then the AR spectrum can be computed from the resulting transfer function:

$$\begin{array}{c} S_{i}^{B}\left(f\right)=\frac{1}{{\left|1+\sum_{k=1}^{p_{B}}a_{ik}^{B}e^{j2\pi fn}\right|}^{2}} \end{array}$$ (2)

**Fig 5:**
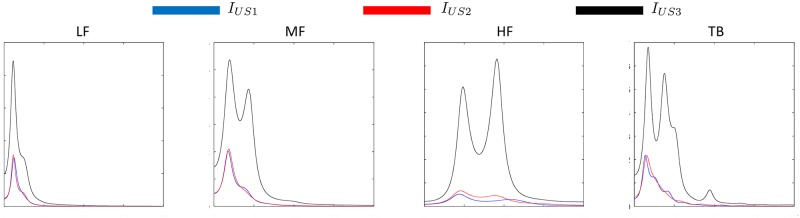
AR spectra for the patches *I*_*US*1_, *I*_*US*2_ and *I*_*US*3_ (in blue, red and black lines respectively) for the four narrowband signals belonging to the ZigZag matrix to signal conversion.

Fig 5 shows an example of the information that the AR spectrum can provide for characterizing tissue. The AR spectra of patches *I*_*US*1_, *I*_*US*2_ and *I*_*US*3_ for LF, MF, HF and TB signals of Fig 4 are displayed. It is possible to observe that for both patches located inside the thyroid (*I*_*US*1_ and *I*_*US*2_) the AR spectra (in blue and red lines respectively) main components are similar for the four narrowband texture signals. For the patch located outside the thyroid (*I*_*US*3_) the AR spectral characteristics are completely different in terms of main frequency components and spectral energy.

These parametrical characteristics can be exploited using the spectral energy of the estimated spectra. Therefore, the features computed in this novel approach are based on band energy ratios computed between the different frequency bands of the AR spectra. These type of AR features have already been used for Heart Rate Variability analysis for extraction of dynamical relationships between sympathetic and parasympathetic activities of the Autonomous Nervous System [23]. Analog to that, we assume that in one US texture the relationship between its different levels of irregularity can provide information for classifying tissue echogenicity.

**Fig 6:**
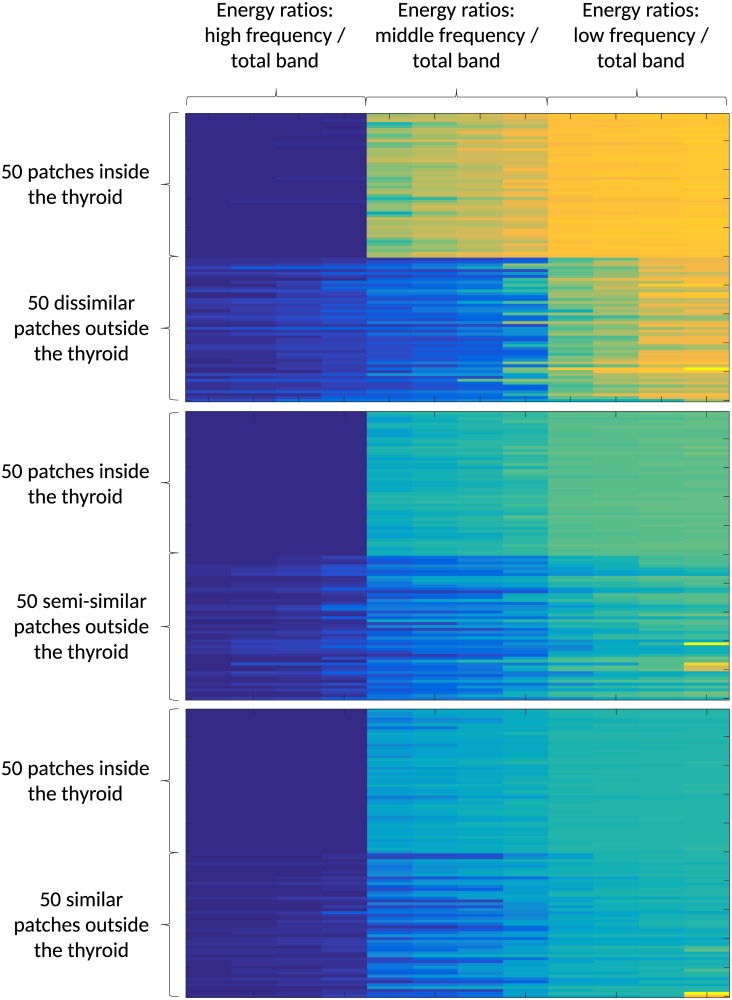
Color-map of the computed features in patches belonging to thyroid and three classes of non-thyroid regions.

Taking into account the number of spectra from the different texture signals belonging to the different conversions, the number of possible spectral energy ratios to be used as potential features is 256. Therefore a procedure of features selection has been performed using an analytic test. First, inconsistent energy ratios were eliminated from the analysis. Then different US images were selected from a US thyroid image dataset (Dataset 1, which will be introduced in the next section). For each image 100 patches (20 × 20 pixels) were selected manually, 50 patches belonging to thyroid and the other 50 belonging to non-thyroid regions. In order to evaluate the ability of a feature to distinguish between thyroid and non-thyroid texture, the patches belonging to regions outside the thyroid were divided in three classes according to the visual level of texture similarity that a non-thyroid region has with respect to thyroid texture: similar, semi-similar and dissimilar. The set of energy ratio features were computed for all the selected patches and the results were plotted using a color-map matrix as shown in Fig 6. Each column of this matrix correspond to an energy ratio feature and allows to visually analyze the ability of an energy ratio feature to distinguish between thyroid and non-thyroid tissues for different levels of similarity degrees.

Following the analysis of the matrix of Fig 6, 30 energy ratios were selected from the previous analysis. According to their characteristics, they can be divided into two types of energy ratios (ER). 4 ERs are computed as the energy of the maximal spectral peak divided by the total spectral energy in a same frequency band and 26 ERs are computed as ratios between total energy of different frequency bands:

$$\begin{array}{c} ER_{1-4}=\frac{\sum_{f=f_{1}}^{f=f_{2}}S_{NUM}\left(f\right)}{\sum_{f=0}^{f=\infty }S_{DEN}\left(f\right)}\;\;\;\;\;\;ER_{5-30}=\frac{\sum_{f=0}^{f=\infty }S_{NUM}\left(f\right)}{\sum_{f=0}^{f=\infty }S_{DEN}\left(f\right)} \end{array}$$ (3)

where *f*_1_ and *f*_2_ are the frequency onset and offset respectively of the main peak and *S*_*NUM*_ and *S*_*DEN*_ are the AR spectra used in the numerator and denominator of Eq (3) and are shown in Table 1 for each of the 30 ERs.

**Table 1:** Spectra used in the numerator (*NUM*) and denominator (*DEN*) of Eq (3) for computing the 30 energy ratio features.

*Features*_1−15_	*NUM*	*DEN*	*Features*_16−30_	*NUM*	*DEN*
*ER*_1_	$S_{1}^{HF}$	$S_{1}^{HF}$	*ER*_16_	$S_{2}^{LF}$	$S_{2}^{HF}$
*ER*_2_	$S_{1}^{MF}$	$S_{1}^{MF}$	*ER*_17_	$S_{2}^{LF}$	$S_{3}^{HF}$
*ER*_3_	$S_{1}^{LF}$	$S_{1}^{LF}$	*ER*_18_	$S_{2}^{LF}$	$S_{4}^{HF}$
*ER*_4_	$S_{1}^{TB}$	$S_{1}^{TB}$	*ER*_19_	$S_{3}^{LF}$	$S_{2}^{HF}$
*ER*_5_	$S_{1}^{HF}$	$S_{1}^{TB}$	*ER*_20_	$S_{3}^{LF}$	$S_{3}^{HF}$
*ER*_6_	$S_{2}^{HF}$	$S_{2}^{TB}$	*ER*_21_	$S_{3}^{LF}$	$S_{4}^{HF}$
*ER*_7_	$S_{1}^{MF}$	$S_{1}^{TB}$	*ER*_22_	$S_{4}^{LF}$	$S_{2}^{HF}$
*ER*_8_	$S_{2}^{MF}$	$S_{2}^{TB}$	*ER*_23_	$S_{4}^{LF}$	$S_{3}^{HF}$
*ER*_9_	$S_{2}^{LF}$	$S_{2}^{TB}$	*ER*_24_	$S_{4}^{LF}$	$S_{4}^{HF}$
*ER*_10_	$S_{3}^{LF}$	$S_{3}^{TB}$	*ER*_25_	$S_{1}^{MF}$	$S_{2}^{HF}$
*ER*_11_	$S_{4}^{LF}$	$S_{4}^{TB}$	*ER*_26_	$S_{1}^{MF}$	$S_{3}^{HF}$
*ER*_12_	$S_{1}^{LF}$	$S_{2}^{HF}$	*ER*_27_	$S_{1}^{MF}$	$S_{4}^{HF}$
*ER*_13_	$S_{1}^{LF}$	$S_{3}^{HF}$	*ER*_28_	$S_{2}^{MF}$	$S_{2}^{HF}$
*ER*_14_	$S_{1}^{LF}$	$S_{4}^{HF}$	*ER*_29_	$S_{2}^{MF}$	$S_{3}^{HF}$
*ER*_15_	$S_{2}^{LF}$	$S_{1}^{HF}$	*ER*_30_	$S_{2}^{MF}$	$S_{4}^{HF}$

The feature extraction algorithm was fully implemented in Matlab R2015b and executed on a PC with a CPU operating at 2.60 GHz resulting in an execution time of 0.06 seconds for computing the 30 features in one patch.

## Results

This section shows the usability of the proposed approach for US feature extraction. 2D US data from thyroid is used in order to analyze the capabilities of the 30 extracted ER features to differentiate between thyroid and non-thyroid tissue in order to use them for segmenting thyroid.

### Thyroid US data description

Two different real US image datasets have been used to evaluate the proposed approach. The first dataset (in the sequel Dataset 1) has been introduced in [24] and involves six healthy human subjects freehand US images acquired using a Logiq E9 US device with a linear probe and equipped with an electromagnetic tracking system. This database has a total of 675 2D US slices with a 760 × 500 pixels with between 53 and 189 US slices per subject. The second dataset (in the sequel Dataset 2) has been presented in [25] and can be downloaded in http://opencas.webarchiv.kit.edu/?q=node/29. It involves freehand US images of 16 healthy subjects, each acquired also with a GE Logiq E9 system but operated by a different clinician in a different hospital than in the Database 1 case. From this dataset, a total of 1600 slices belonging to the 16 subjects (100 slices per subject) were used with a size of 760 × 1020 pixels per 2D US slice.

For each 2D slice the thyroid was manually segmented by an expert clinician (ground truth) and was then divided into patches of 20 × 20 pixels labelled as thyroid or non-thyroid according to the ground truth. It is important to notice that in both datasets the number of patches belonging to thyroid are less that the ones belonging to non-thyroid. This is because the ground truth was used for the automatic patch labelling and usually in a US image the region of thyroid is smaller than the non-thyroid one.

### Average value differences between thyroid and non-thyroid patches for the selected features

**Fig 7:**
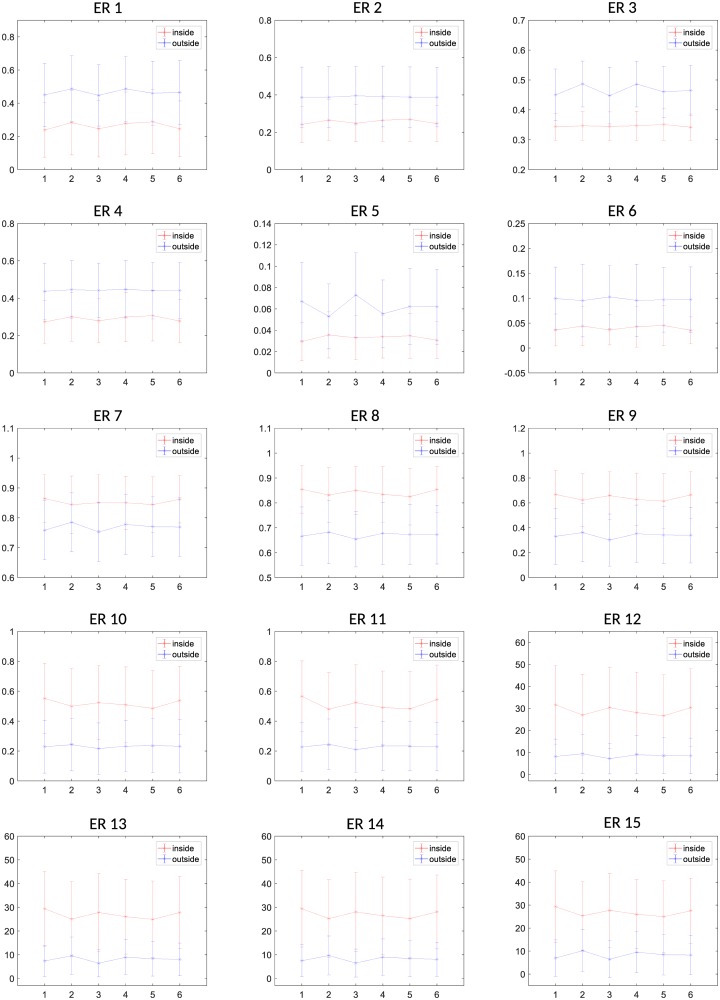
Mean and standard deviation of values of ERs features 1 to 15 of thyroid and non-thyroid patches for the 6 subjects of the Dataset 1.

**Fig 8:**
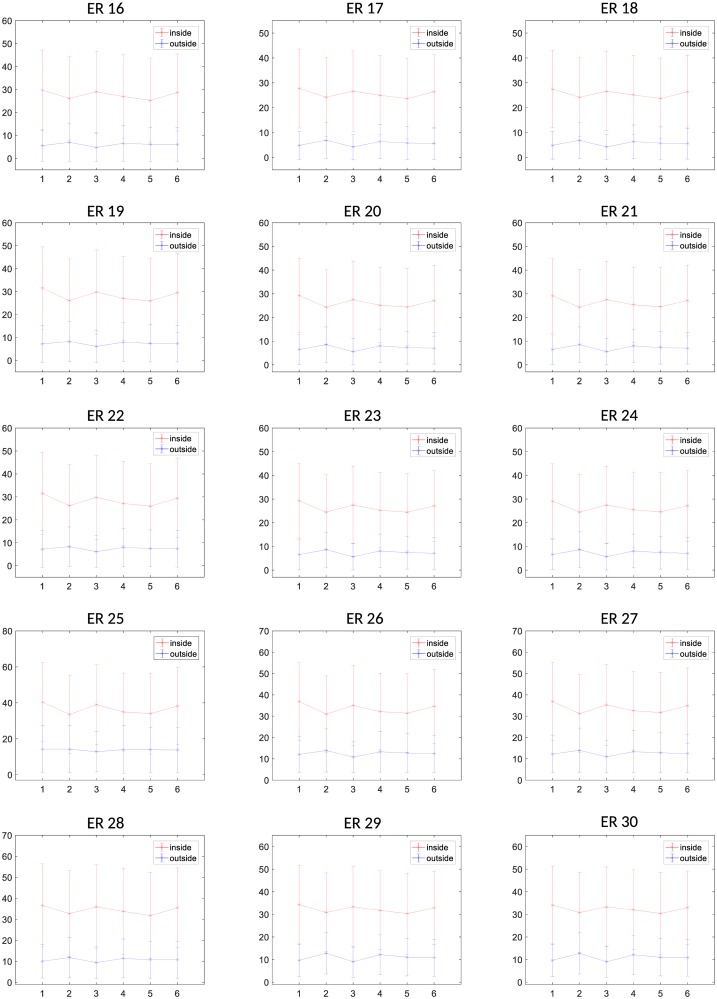
Mean and standard deviation of values of ERs features 16 to 30 of thyroid and non-thyroid patches for the 6 subjects of the Dataset 1.

In order to observe the capacity and suitability of the selected features for distinguishing between thyroid and non-thyroid texture, the average and standard deviation (STD) of the feature values were computed for the six subjects belonging to Dataset 1 (see Figs 7 and 8). It is possible to visualize that for the whole set of selected ER features, the average values are clearly different between thyroid (red) and non-thyroid (blue) tissues. Moreover, in most of the ER features the thyroid and non-thyroid average values do not strongly change from one subject to another one. Concerning the STD, it is possible to observe that some ER features works better than others. This is the case for example of features *ER*_3_ and *ER*_6_ where the STDs inside the thyroid are much smaller than outside the thyroid, what is consistent to the homogeneity of texture inside one healthy organ.

### Features evaluation for thyroid segmentation

The proposed approach have been tested on the 359712 patches of Dataset 1 and on the 1791397 patches of Dataset 2. For each patch, the 30 ERs of Eq (3) were computed and analyzed to see their suitability to distinguish between thyroid and non-thyroid tissues.

**Fig 9:**
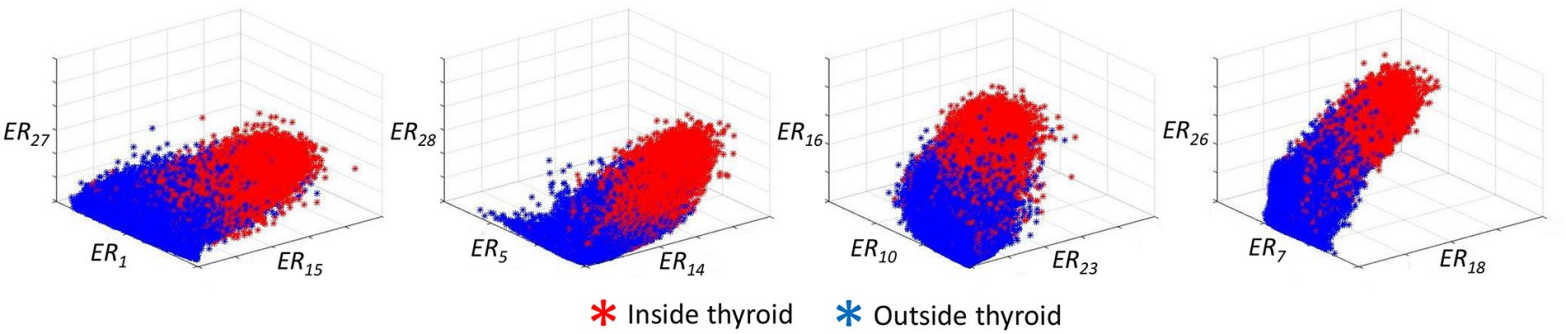
Example of obtained AR spectral energy ratios when the approach is applied to the full set of patches extracted from the thyroid US Dataset 1.

In Fig 9 3D scatters are displayed for 12 ERs (in groups of three features) computed from Dataset 1 clearly showing the differences of these ratio values for thyroid (in red) and non-thyroid (in blue). This confirms that the AR characterization of US texture is well suited to obtain features that are able to be used for classification of thyroid tissue.

To evaluate the performances of our approach, the computed ER features were used to segment the thyroid. Because our goal is to show the usability of the extracted AR features for texture characterization in US images, complex classification procedures were avoided. Therefore only a simple K-Means algorithm for clustering the patches as thyroid or non-thyroid using the 30 features computed in both datasets was used in this work. As an unsupervised classification for thyroid segmentation, this automatic labelling was then used in each US image to separate the patches belonging to that image as thyroid and non-thyroid.

**Fig 10:**
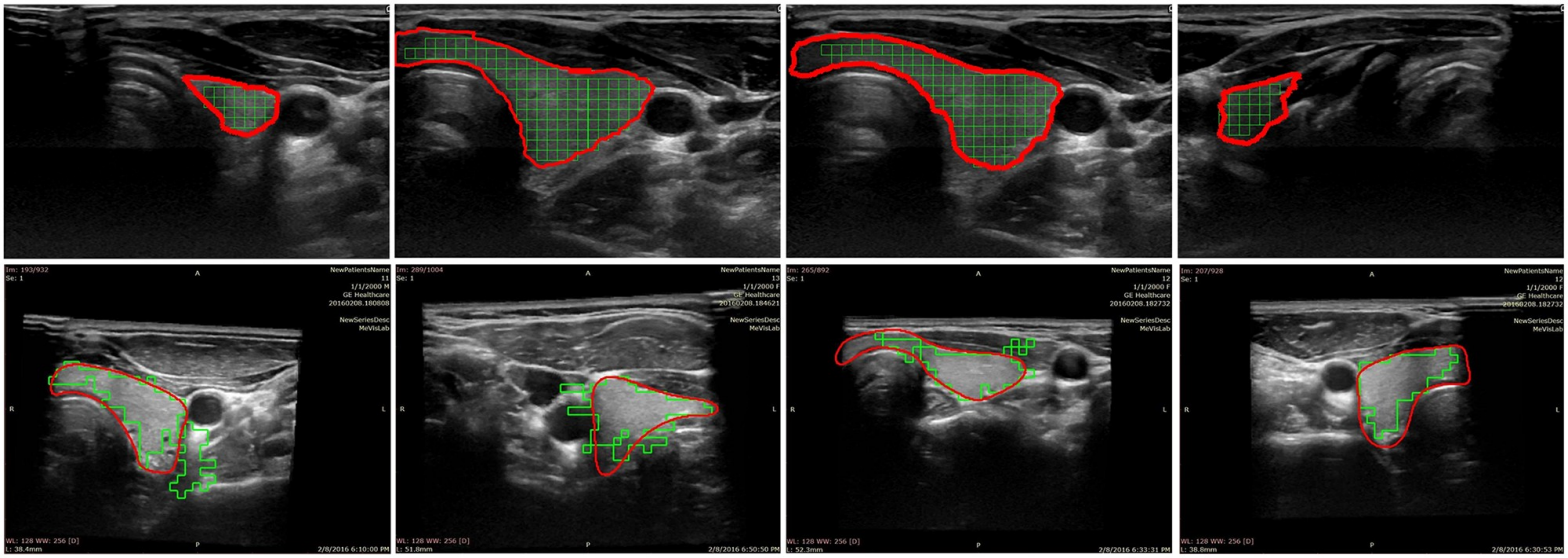
Examples of thyroid segmentation using the proposed approach and comparison with the ground truth.

Fig 10 shows some example results of the thyroid segmentation for eight slices: four examples for correct segmentation (first row) and four involving some false positives (second row). In solid red line the ground truth is displayed and the green squares correspond to the 20 × 20 patches that were classified as thyroid by applying our approach to each one of the US images. We displayed US images belonging to different subjects and also to different positions of the 2D slices with respect to the thyroid volume.

In order to globally evaluate our approach with both datasets, the Dice coefficient (DC) is computed from all the 2D segmentations for each Dataset. Additionally, the sensitivity (SE) and specificity (SP) are also computed. The approach obtains a DC of 89.66% with a SE of 0.95 and a SP of 0.70 for the Dataset 1 and a DC of 86.89% with a SE of 0.89 and a SP of 0.62 for the Dataset 2.

In order to analyze the significance of our results, the proposed approach was compared with other thyroid segmentation methods proposed in the literature. For that the comparison results reported in [8] and in [9] are used.

In [8] five thyroid segmentation algorithms are compared using ten subjects, where six of them are taken from Dataset 1 used in this work. The algorithms that this work compares in terms of Dice Coefficient are Active Contour Without Edges (ACWE), Graph Cut (GC), Pixel-Based Classifier (PBC), Random Forest Classifier (RFC) and Convolutional Neural Network (CNN). The first three are semi-automatic requiring different levels of interaction with the operator and the other two are automatic. The results of this comparison are displayed in Table 2 showing that our approach outperforms the other five algorithms.

**Table 2:** Comparison of the proposed approach in terms of Dice Coefficient using the Dataset 1 with algorithms compared in [8].

ACWE	GC	PBC	RFC	CNN	this work
80.53%	74.52%	66.68%	85.53%	87.22%	**89.66%**

In [9] an algorithm based on Iterative Random Walks and Random Forest (IRWRF) was evaluated with the Dataset 2 used in this work. They have compared their approach with four other algorithms presented in the literature: Echogenicity-based Quantization (EBQ), Joint Classification-Regression (JCR), RBF Neural Network (RBF), and Feedforward Neural Network (FNN). However the reported results for the other algorithms do not use the same dataset. Despite this fact, we display the results of this comparison in Table 3. The algorithm were compared in terms of DC, SE and SP.

**Table 3:** Comparison of the proposed approach using Dataset 2 with five algorithm results reported in [9].

	IRWRF	EBQ	JCR	RBF	FNN	this work
DC	85.4%	83.9%	47.9%	51.2%	40.0%	**86.9%**
SE	98.9%	95.5%	56.4%	87.4%	47.3%	**89.0%**
SP	92.3%	88.9%	92.6%	56.0%	86.4%	**62.0%**

## Conclusions and discussions

In this work a novel approach for ultrasound image feature extraction was presented. The approach is based on characterizing ultrasound texture through parametrical modelling. The image was transformed into a signal, which was decomposed in several dynamics representing different aspects of the texture. We showed that features consisting on frequency band based energy ratios between the different signal dynamics contain valuable information about texture and can be useful for US image texture classification.

The usability of the proposed approach was demonstrated in US thyroid segmentation. The 30 extracted AR features computed from energy ratios of the parametrical AR spectra obtain very good and reproducible results for differentiating thyroid and non-thyroid regions in US images. Using a simple K-Means procedure we demonstrated that thyroid patches were successfully clustered for thyroid segmentation. The approach was evaluated with two datasets and compared with ten other algorithms proposed in the literature, obtaining Dice Coefficients over 85% and outperforming other methods.

We strongly believe that this approach can be used in a variety of US applications, not only for segmentation, but also for data comparison, pattern recognition and possibly others. The presented research contribution and scientific innovation could lead to an objective characterization and differentiation of tissues in US, but likely also be used for other bio-medical imaging methods.

One of the drawbacks of the proposed approach is that edges between two tissues are prone to segmentation errors. This is due to the patch approach that we have used. In order to deal with this problem the next steps is to implement a *space-variant* AR modelling, analogue to the time-variant version generally used for non-stationary signal processing. This not only should deal with tissue border problems but also should allow to process *signal trajectories* in a volume in order to perform voxel characterization.

The main focus of this paper was not on thyroid segmentation but on analyze the usability of features that have never been used in the literature for US image analysis. These novel proposed features, even if they are linear, they have obtained interesting results in signal processing in application fields such as biosignal processing or tool wear monitoring. We wanted to analyze how this type of features could work for extracting characteristics from US images. However, in the near future many aspect of this research should be treated in order to think in clinical significant results for US texture characterization. First, a more exhaustive analysis and optimization needs to be performed for AR features selection. It is not only necessary to revise the spectral energy based features but also to see how other AR features (such as pole-based or space variant features) can be used for US texture characterization. Second, a next step should focus on analyze how AR features together with nonlinear features (such as higher order statistics or entropy-based features) and a deep learning procedure can work not only for thyroid segmentation but also for thyroid lesion classification. Finally, in order to show clinical significance, further research is required in order to see the behaviour of the proposed features in larger datasets involving unhealthy thyroids, different US acquisition parameters, different probes and US devices. This is the main next step for our approach.
